# Some of the Causes of Failure to Cure Chronic Suppuration of the Middle Ear

**Published:** 1888-04

**Authors:** J. H. Smith

**Affiliations:** Dallas, Texas


### SOME OF THE CAUSES OF FAILURE TO CURE CHRONIC SUPPURATION OF THE MIDDLE EAR.

*By J. H. Smith, M. D., Dallas, Texas.*

For Daniel's Texas Medical Journal.

There is perhaps, no chronic disease so annoying and hard to control, as that accompanying a neglected or improperly treated case of acute suppurative otitis media.

There is a popular idea prevailing among the laiety, and I am sorry to say, among some physicians, that is responssble to a large extent for a chronic suppurative condition of the middle ear. This idea is that if the ear is let alone during or after the acute attack, that it will get well of itself, or that the child, (for it is in children we find this condition most frequently existing), will out-grow it. That occasionally an acute suppurative inflammation subsides, and the discharge ceases, I do not deny, but such cases are so rare an exception to the rule, that they could hardly be said to amount to anything in statistics.

A casual examination of the nares, posterior nasal cavity and upper pharynx will show the cavities lined with the same continuous mucous membrane;and it is a well known observation that in every attack of acute suppurative inflammation of the middle ear, this entire surface of mucous membrane is in a high state of congestion. Hence arises the common expression of having an earache from a bad cold.

All the distressing symptoms accompanying an acute ear-ache, that can hardly be described except by one who has suffered, become more and more aggravated, until the formation of pus ensues, which bursting through the membrane tympani, pours itself out into the external auditory canal, and bringing grateful relief to a suffering that has become well-nigh intolerable. At this stage of the inflammation, the ear is subjected to a series of syringing, applications of liquids and powder, etc., until the patience, both of physicians and patient is exhausted, and they are disappointed in not seeing any permanent results from treatment. The pain and swelling may have subsided, but there remains behind, in spite of faithful and well-meaning efforts, a suppurative condition of the middle ear cavity, which soon assumes the chronic form, accompanied perhaps by a mass of granular tissue protruding through the perforation of the membrane, into the canal. In this condition they are advised to seek some aurist, and their history shows that they have borne with this class of ear trouble through many years, from early childhood to manhood or womanhood. Theobject of this paper is to direct attention to the treatment of a locality often neglected in handling aural patients, viz: post nasal and pharyngeal cavities. Hypertrophy of the turbinated bones often co-exists with a chronic inflammation of these cavities, and pressure from these hypertrophies, forms an important factor in the production and continuation of local inflammation in this region.

A brief extract of a few cases from my case-book, will explain what I mean.

James M., aged 30, white, consulted me Nov. 25th, 1887, with a history of chronic suppurative otitis media, of fifteen years standing. Membrane tympani of left ear perforated in lower inferior quadrant, with auditory canal bathed in a profuse purulent discharge, which was very offensive. Says he had scarlet fever when fifteen years old, and resulted in this ear trouble. Has been treated by several physicians, who would succeed in stopping the discharge for a while, when it would re-appear. Hears well on right side. On left side, hearing reduced to five inches. An examination of the nose and upper pharyngeal space, revealed a chronic hypertrophied condition of the turbinated bones; with thickening of the mucous lining of the pharynx.

Treatment—Ear was wiped out with dry absorbent cotton, and the canal filled with peroxide of hydrogen. This was forced into the middle ear through the perforation in the membrane, by causing the patient to turn his head downward and to the right, and pressure being made at the external opening of the canal in such a manner as to force the fluid into the middle ear. Any residue was allowed to escape and the ear afterwards thoroughly dried. The nasal cavity was thoroughly cleansed with a warm alkaline spray, and the hypertrophied tissue upon the turbinated bones was touched with chromic acid. This latter application producs an open wound, which upon healing, contracts by cicatrization, and causes the hypertrophy to disappear. The post-nasal cavity as well as the upper pharynx receiving at the same time appropriate treatment with astringents.

Under this treatment the ear discharge soon ceased, nor has it returned at this date.

Case II. Mary M., aged 11, has a history of chronic suppuration of the right ear since early childhood. The watch test showed almost entire loss of hearing on right side, except by close pressure of the watch to the ear. Hearing on left side slightly impaired. Examination of nose revealed a thickened and swollen condition of the turbinated bones, which were bathed in a muco-purulent discharge. Tonsils also hypertrophied. The method of treatment was the same as in case 1, particular attention being directed to the enlarged turbinated bones, which projected so far into the nasal cavity, as to enable me to trim their edges, with my long nasal scissors, instead of using the chromic acid. Valsalva's method was used in inflating the eustachian tubes, by which means I was enabled to force a considerable amount of purulent matter mixed with epithelial debris, into the external canal, from whence it was easily removed. Two months treatment sufficed to effect a cure, besides restoring the hearing to a considerable degree in the affected ear.

Case III. Helen A. aged 13, was brought to my office with a history of a discharge from both ears since she was a baby. Suffers no particular pain, but finds considerable trouble in keeping her ears clean; says she can smell is well as taste the odor arising from the putrid discharge; also says her breathing through the nose is irregular and interrupted. An aural examination displays both membrane tympani, destroyed except a narrow outer rim. The left nostril is nearly entirely closed from an hypertrophied turbinated bone, the right occluded with a polypus. The tonsils were also enlarged. The ears cleaned with per-oxide of hydrogen, and dried, then boracic acid blown into the tympanum. The nasal polypus was removed, tonsils trimmed, and the hypertrophy of the turbinated bone treated. Though still under treatment, the aural discharge has ceased and the nose and pharynx once more assuming a healthy look. Her voice is losing that nasal twang so often noticed in occlusion of the nasal passages, and general improvement with the exception of her hearing is very satisfactory.

My first examination of ear cases applying to me, I do not con sider complete until I have made a thorough examination of the upper air-passage, including the nares, post-nasal space and pharynx. This I regard as of prime importance, in this portion of the South, where we have to deal with all varieties of catarrhal affections occurring in the extremely young as well as the more advanced in life. I now never think of placing a case of chronic suppuration of the ear under treatment without a thorough treatment of the nose and throat, if Ifind the above diseased conditions existing. Any success I may have attined in handling many of these cases who have been under the care of other physicians, I attribute to the detailed attention to the nose and throat, which I have found to be more or less affected in nearly every case of aural trouble of the chronic suppurative variety. A mere checking the discharge from the ear, does not cure. There always remains more or less deafness when the discharge ceases, which can be greatly relieved and sometimes entirely cured, by a proper medication of the upper air passages, together with the use of the Valsalva method.

